# Video-Based Sign Language Recognition via ResNet and LSTM Network

**DOI:** 10.3390/jimaging10060149

**Published:** 2024-06-20

**Authors:** Jiayu Huang, Varin Chouvatut

**Affiliations:** Department of Computer Science, Faculty of Science, Chiang Mai University, Chiang Mai 50200, Thailand; jiayu_huang@cmu.ac.th

**Keywords:** sign language recognition, deep learning, ResNet, LSTM

## Abstract

Sign language recognition technology can help people with hearing impairments to communicate with non-hearing-impaired people. At present, with the rapid development of society, deep learning also provides certain technical support for sign language recognition work. In sign language recognition tasks, traditional convolutional neural networks used to extract spatio-temporal features from sign language videos suffer from insufficient feature extraction, resulting in low recognition rates. Nevertheless, a large number of video-based sign language datasets require a significant amount of computing resources for training while ensuring the generalization of the network, which poses a challenge for recognition. In this paper, we present a video-based sign language recognition method based on Residual Network (ResNet) and Long Short-Term Memory (LSTM). As the number of network layers increases, the ResNet network can effectively solve the granularity explosion problem and obtain better time series features. We use the ResNet convolutional network as the backbone model. LSTM utilizes the concept of gates to control unit states and update the output feature values of sequences. ResNet extracts the sign language features. Then, the learned feature space is used as the input of the LSTM network to obtain long sequence features. It can effectively extract the spatio-temporal features in sign language videos and improve the recognition rate of sign language actions. An extensive experimental evaluation demonstrates the effectiveness and superior performance of the proposed method, with an accuracy of 86.25%, F1-score of 84.98%, and precision of 87.77% on Argentine Sign Language (LSA64).

## 1. Introduction

Sign language is a vital bridge for communication between deaf people and non-deaf people. Speakers will express their thoughts through various body movements and expressions, but few people in the some population can understand sign language [1,2,3]; the goal of sign language recognition is to enhance a computer’s understanding of human sign language, thereby promoting barrier-free communication between deaf and hearing individuals.

Sign language is a set of compound actions that includes hand movements, body postures, and facial expressions to jointly display lexicon [4]. Most sign language meanings are explained by hand movements and body postures; some emotional information can be expressed through facial expressions, such as using facial expressions to express joy, anger, sorrow, and happiness, and some can use facial expressions to distinguish the same gesture from expressing different meanings.

**Figure 1:**
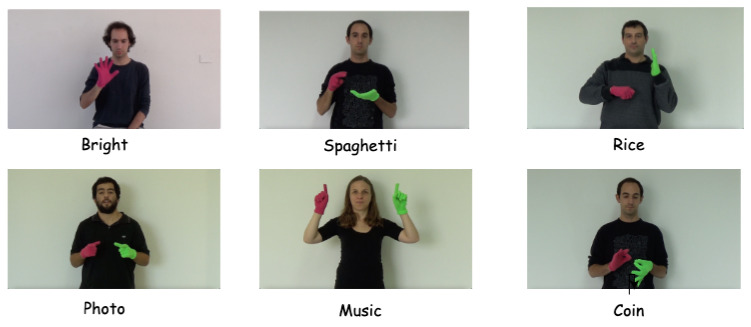
The diagram shows the keyframes extracted from Argentine Sign Language videos with different words, where different sign words have different action expressions.

Video-based sign language determines a continuous image frame with a temporal concept. The movements of gestures constitute the basic components of sign language [5]. Sign language can be divided into static sign language and dynamic sign language based on its state [6]. Static sign language mainly includes basic letters and some basic traffic signs; it is represented by the shape and direction of hands and fingers, which constitute the basic actions of sign language words [7]. Dynamic sign language consists mainly of hand movements and facial expressions and therefore requires video streaming to record these combinations of movements as shown Figure 1. For sign language recognition, all we do is recognize the sign language movements in a video stream.

There are different forms of sign language expression in different countries, and the correlation between sign language and spoken language is complex. The formation of sign language is, to a large extent, similar to the local living habits [8,9,10]. For the same language, different environments have different forms of sign language expression, such as American Sign Language (ASL) and British Sign Language (BSL), which are both in English but have different forms of sign language articulation. During sign language recognition tasks, the recognition methods are generic and require an annotation of the dataset, followed by the use of algorithms to recognize and classify the data.

Traditional sign language recognition methods build temporal models by manually extracting features, and the temporal models used, including Hidden Markov Models [11], Conditional Random Fields [12], Dynamic Time Warming [13], and manually feature extraction, have been reliant on the experience of designers, and the process of temporal modeling is cumbersome, with no breakthroughs in many years. Researchers have used Convolutional Neural Networks (CNNs) to exploit hand-shape features with good results [14]. Sign language words are made up of video sequences, and extracting features using 2D-CNN networks loses temporal information. Deep neural networks can extract spatio-temporal features of videos, which is a significant breakthrough in behavior recognition and provides new insights for sign language recognition.

Although the methods mentioned above can perform sign language recognition, there are still some problems. With the increase in data volume and the number of network layers, neural networks are prone to a series of issues that affect their stability, such as gradient explosion and gradient vanishing [15]. In time series, not only should the short-term feature space be considered, but the long-term feature space cannot be ignored. Long-term time series can highlight global features and better capture the correlation between sequences in sign language recognition.

In real life, using deep learning for sign language recognition not only requires real-time recognition but also highlights the effectiveness of the model; large models and 3D convolutional neural networks can yield good experimental results, but when deployed on devices with limited graphics processing unit (GPU) resources, such as smartphones with smaller memory and personal computer terminals, resource consumption costs need to be considered. ResNet 18 is used as a backbone, and we take advantage of its following advantages: (1) it has fewer layers and is relatively easy to deploy; (2) it is also possible to effectively alleviate the problem of overfitting utilizing its residual network; and (3) in the process of knowledge transfer, its hyperparameters are easy to modify to obtain acceptable recognition results.

In this paper, we propose a framework for video-based sign language recognition using ResNet [16] and LSTM. The framework first uses the ResNet network to extract sign language features, obtain the sign language feature space, and then feeds the features into the LSTM to obtain long-term sequence features. Finally, a fully connected layer is used for classification. The notable contributions of this study can be summarized as follows:

- Word-level sign language recognition from sequential video files in Argentine sign language is performed.
- A new end-to-end fusion ResNet and LSTM network is proposed for video-based sign language sequence recognition. The network extracts key sign language features through residual networks, captures the long-term relation of the video sequence, and achieves a better classification performance.
- The pre-trained recognition method used in this research improves in the recognition accuracy and training time in our model generation.
- The motion recognition of word-level sign language can be achieved with higher performance compared with other methods, as shown in the comparison table in the results and discussion section.

## 2. Related Work

The research on sign language recognition has been widely carried out both domestically and internationally, covering both theoretical and practical aspects and many technical tasks. Sign language recognition based on different data processing technology can be summarized as follows.

Sensor devices can be used to obtain gesture change signals and upper limb movement trajectories for modeling and achieving sign language translation. In 1983, Grimes et al. [17] were the first to use data gloves for sign language recognition research and achieved the recognition of American Sign Language. Subsequently, more and more sign language recognition researchers are using data gloves for research on sign language recognition. Oliveira et al. [18] used two-handed data gloves to capture sign language movements and fed them into neural networks for recognition, achieving the recognition of English words. Lin et al. [19] used cameras to obtain data on people wearing colored gloves and performed data preprocessing, such as color segmentation, on these image data. Although sensor-based sign language recognition has made significant progress, these devices require sign language performers to comply with specific wearing requirements, making the entire process cumbersome. Traditional methods such as image processing, sequence, and classification algorithms are mainly used to achieve sign language recognition. Maharni et al. [20] proposed a gesture action classification system based on support vector machines. Liu et al. [21] used a K-value nearest neighbor method for gesture recognition that measures the distance between different feature values for classification. Zhang et al. [22] proposed a model that combines DTW (dynamic time warping) and an HMM (hidden Markov model) for recognizing continuous sign language videos. The experimental results showed that this method can effectively reduce word error rates. Although traditional methods for sign language recognition have achieved certain results in accuracy, the limitations of manual computation and the complexity of gesture actions result in the use of manually set features greatly increasing the workload of sign language recognition. Therefore, more and more researchers are beginning to invest in sign language recognition based on deep learning.

With the development of deep learning, researchers are using it for sign language recognition. Koller et al. [23] achieved high recognition rates on the PHOENIX-2014 dataset by combining CNNs (convolutional neural networks) with an HMM for continuous sign language sentences. Considering the timing issue of sign language videos, Tran et al. [24] extended traditional 2D convolution to 3D convolution to obtain temporal features between video frames. Pigou et al. [25] captured the hand features of the human body based on a CNN structure and constructed an Italian Sign Language recognition system, which achieved an accuracy of 91.7% for recognizing Italian Sign Language datasets. Cui et al. [26] used CTC (connectionist temporal classification) to label time segments and combined a CNNs and RNNs (recurrent neural networks) to improve the recognition rate of sign language videos so as to extract advanced features from video time series information.

The Long Short-Term Memory (LSTM) network, as introduced by Hochreiter and Schmidhuber [27], represents a specialized Recurrent Neural Network (RNN) model distinguished by its unique structural design, which is adept at mitigating long-term dependency issues. Unlike conventional RNNs, the LSTM inherently possesses the ability to retain early information, incurring no additional computational cost for this default behavior. The LSTM achieves this by employing four distinct neural network layers that interact in a specialized manner. These layers are strategically designed to facilitate the learning of feature information in sequences. The incorporation of forgetting gates, memory gates, and output gates allows the LSTM to selectively control the retention and transmission of sequence information. The cell state, serving as a repository of information, is utilized to store and transmit relevant data to subsequent LSTM units. This intricate process culminates in the reflection of the learned information into the cell state and output, enabling the effective handling of sequential data with long-term dependencies. Mali et al. [28] used MediaPipe as a whole and LSTM modules to recognize the sign language of people with disabilities. MediaPipe Holistic integrates pose, hand, and facial key points with precision levels and is used due to its low latency and high tracking accuracy in real-world scenarios. It then uses an LSTM module for sign language classification and recognition. A dynamic sign language recognition method based on an improved LSTM model was proposed by [29], using leap motion to collect sign language and then using the LSTM network with an attention mechanism for dynamic sign language recognition to achieve good results. Li et al. [30] proposed a physical information neural network model combined with physical laws to predict the limit of fluid mechanics, with the baseline being the use of an LSTM for prediction. This interdisciplinary fusion provides an innovative method. Li et al. [31] studied uncertainty and proposed a fusion model using LSTM and Monte Carlo methods to explore its uncertainty. In the absence of uncertainty, good results were achieved.

## 3. Methods

### 3.1. Notation

A sign language dataset with *L* training example labels is denoted by $T_{s}=\left\{X_{i},Y_{i}\right\}$ , where $X_{i}\in R^{C\times T\times H\times W}$ ; *C* is the channels of frame; *T* is the number of frames; *H* and *W* are the height and width of the frame, respectively; and $Y_{i}\in R^{k}$ is a label of *K* classes. Among them are $\left\{x_{i}\in X,y_{i}\in Y\right\}$ . We also consider a complementary source domain set of sign language data denoted by $D_{i}={\left\{D_{i},M_{i}\right\}}_{i}^{N}=1$ . Similarly, $D_{i}$ is an RGB video. $M_{i}$ represents the label sequence corresponding to $D_{i}$ .

### 3.2. Overview

**Figure 2:**
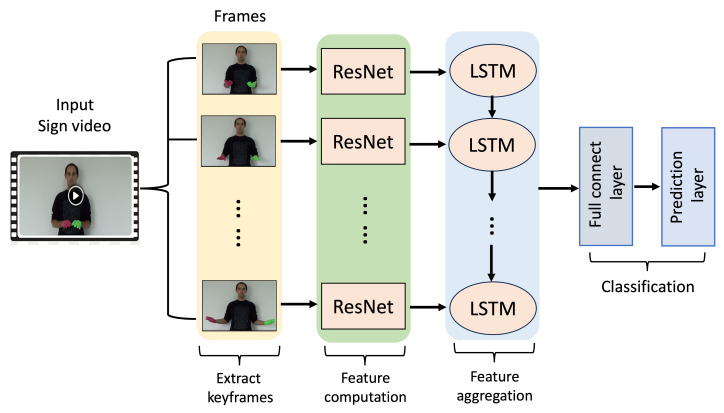
The overview of the proposed model based on ResNet and LSTM.

We noticed that with the increase in the number of sign languages and the number of layers in deep learning networks, the model may suffer from gradient overfitting and exploding, which can lead to decreasing the robustness and recognition accuracy of the model; time cost as a part of the model training also needs to be considered. Long sequence features can better capture the correlation between time series. To address the aforementioned issues, we propose using a fusion network of ResNet and LSTM to train a sign language recognition model. As the network layers increase, the ResNet network can effectively solve the problem of gradient explosion and obtain better time series features. The LSTM can obtain long sequence features. The specific process of this method is shown in Figure 2. The video is preprocessed by dividing it into video clip with 16 frames, each video clip is fed into the ResNet network for feature extraction to obtain a feature space. Secondly, the learned feature space is used as the input of the LSTM to obtain long-term features. Finally, the video is classified through a fully connected layer, and the prediction results are output.

### 3.3. Residual Convolution Network

The depth of the network is crucial to the performance of the model. When the number of network layers is increased, the network can extract more complex feature patterns. Therefore, theoretically, better results can be achieved when the model is deeper. However, is the performance of a deeper network necessarily better? The experiment found that deep networks have a degradation problem: as the depth of the network increases, the accuracy of the network saturates or even decreases. We know that deep networks have the problem of vanishing or exploding gradients, which makes it difficult to train deep learning models. But now there are some technological means such as Batch normalization for alleviating this problem. Therefore, the problem of deep network degradation is very surprising.

**Figure 3:**
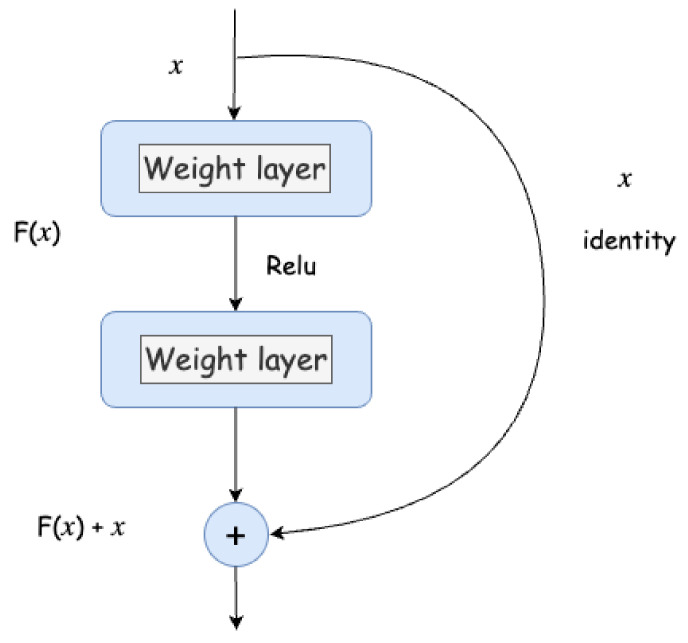
The diagram shows a ResNet block: *x* is the input data, the fitting function of the model is F(x) , and the expected latent mapping is F(x)+x .

He et al. [16] proposed residual learning to solve the degradation problem. For a stacked-layer structure (composed of several layers), when the input is *x*, the learned features are denoted as H(x) . Now, we hope that it can learn residuals F(x)=H(x)−x so that the original learned features are H(x) . The reason for this is that residual learning is easier than directly learning the original features. Figure 3 shows the design of the residual block, where *x* is the input data, F(x) is the fitting function of the model, and F(x)+x is the expected latent mapping. When the residual F(x) is 0, the stacked layer only performs identity mapping; at least the network performance will not decrease. In fact, the residual will not be 0, which will enable the stacked layer to learn new features based on the input features, thus achieving a better performance. This is somewhat similar to a “short circuit” in a circuit, so it is a type of short connection. The residual unit can be represented as follows:

(1) $$\begin{array}{c} y_{l}=h\left(x_{l}\right)+F\left(x_{l},W_{l}\right) \end{array}$$

(2) $$\begin{array}{c} x_{l+1}=f\left(y_{l}\right) \end{array}$$

Among these, $x_{l}$ and $x_{l+1}$ represent the input and output of the *l*- th residual unit, respectively. Note that each residual unit generally contains a multi-layer structure. F(x) is a residual function that represents the learned residual, while $h\left(x_{l}\right)=x_{l}$ represents the identity mapping, and f(·) is a ReLU activation function. Based on the above equation, we obtain the learning features from the shallow *l*- th to *L* deep layers as follows:

(3) $$\begin{array}{c} x_{L}=x_{l}+\sum_{i=l}^{L-1}F\left(x_{i},W_{i}\right) \end{array}$$

$\sum_{i=l}^{L-1}F\left(x_{i},W_{i}\right)$ denotes features from layer *l* to *L*-1. In using the chain rule, the gradient of the reverse process can be obtained:

(4) $$\begin{array}{c} \frac{\partial loss}{\partial x_{l}}=\frac{\partial loss}{\partial x_{L}}\cdot \frac{\partial x_{L}}{\partial x_{l}}=\frac{\partial loss}{\partial x_{L}}\cdot \left(1+\frac{\partial }{\partial x_{l}}\sum_{i=l}^{L-1}F\left(x_{i},W_{i}\right)\right) \end{array}$$

The first factor $\frac{\partial loss}{\partial x_{l}}$ in the formula represents the gradient *L* reached by the loss function, while the 1 in parentheses indicates that the short-circuit mechanism can propagate the gradient without loss, while the other residual gradient $\frac{\partial }{\partial x_{l}}\sum_{i=l}^{L-1}F\left(x_{i},W_{i}\right)$ needs to pass through a layer with weights, and the gradient is not directly transmitted. The residual gradient is not always −1, and even if it is relatively small, the presence of 1 will not cause the gradient to disappear. So residual learning will be easier.

In using residual modules to increase the network depth, the neural network in sign language video recognition tasks can retain both low-level and deep features without causing excessive repetitive learning. This can obtain the optimal feature representation to improve the accuracy of sign language recognition and the representation of semantic information. For sign language video recognition models, excessively deep network layers may focus too much on details and overlook the overall picture. Therefore, this study used ResNet18 as the sign language feature extraction model.

### 3.4. LSTM Network

**Figure 4:**
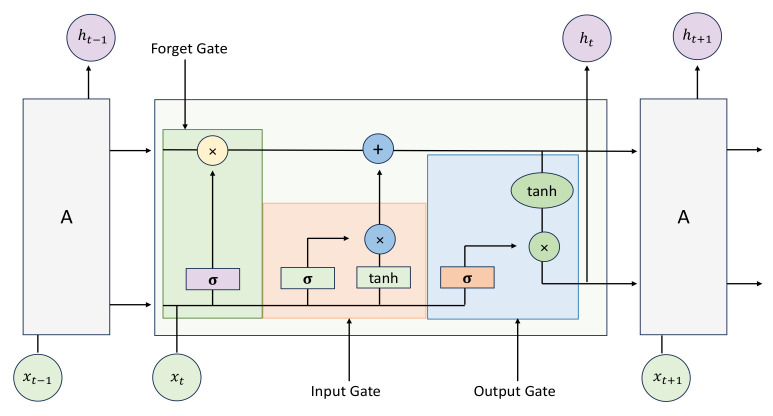
The diagram shows the structural information of the LSTM, where *c* represents the cell state and is used to store the current state information and transmit it to the next LSTM. *x* represents the output information of the previous time, which is the output gate. $f_{t}$ represents the forgetting gate, $i_{t}$ represents the memory gate, and $c_{t}$ represents the information that needs to be updated.

The sign language recognition task requires attention to the spatial and temporal features of the video. ResNet can extract a large amount of useful spatial feature information, but it has some shortcomings in extracting temporal feature information. The RNN model can effectively handle variable-length data and model it, with a natural time depth for extracting temporal features. However, traditional RNN structures may cause network gradients to disappear or explode during model training due to long time spans, while sign language recognition tasks require long-term dependence on network timing. To address these issues, this study adopted LSTM networks for the temporal modeling of sign language recognition. As illustrated in Figure 4, the LSTM network consists of three gates, an activation function, and a memory unit.

(5) $$f_{t}=\sigma \left(W_{xr}*x_{t}+W_{hr}*H_{t-1}^{k}+b_{r}\right)$$

(6) $$i_{t}=\sigma \left(W_{xi}*x_{t}+W_{hi}*H_{t-1}^{k}+b_{i}\right)$$

(7) $$g_{t}=tanh\left(W_{xg}*x_{t}+W_{hg}*H_{t-1}^{k}+b_{g}\right)$$

(8) $$o_{t}=\sigma \left(W_{xo}*x_{t}+W_{ho}*H_{t-1}^{k}+b_{o}\right)$$

(9) $$c_{t}=f_{t}c_{t-1}+i_{t}g_{t}$$

(10) $$h_{t}=o_{t}tanh\left(c_{t}\right)$$

Here, σ is the sigmoid function, tanh is a hyperbolic tangent function, and *W* is the weight matrix. Forgetting gate $f_{t}$ indicates how much information needs to be discarded and saved in the previous moment, while the remaining useful information is used at the current moment to handle the problems of gradient vanishing and exploding. Input gate $i_{t}$ is used for filtering new memory expressions by discarding unnecessary information and retaining new useful information. Add the memory retained at the previous time in the network to the memory retained at the current time to obtain a new memory. The tanh layer $g_{t}$ generates a set of candidate values, which will be added to the storage unit if the input gate allows. According to (7), update the storage unit $c_{t}$ based on the output of the forget gate $f_{t}$ , input gate $i_{t}$ , and new candidate value $g_{t}$ . In Formula (8), the output gate $o_{t}$ controls the state and memory information of the hidden state. The new memory $c_{t}$ is calculated by adding the memory retained at the previous time in the network to the memory retained at the current time, as shown in Formula (9). Operate on the new information obtained through the tanh function with the new memory $c_{t}$ to obtain the current required information for output $h_{t}$ .

### 3.5. Pre-Training

In this study, we used pre-trained models to reduce the training time and trained on the ImageNet [32] dataset using ResNet. The default learning rate schedule starts at 0.1 and decays by a factor of 10 every 30 epochs. We set the network to 18 layers and performed scratch training on the same input. The network parameters are shown in Table 1, and the network consisted of 5 convolutional layers. We used batch normalization in each layer and set the batch size to 32. Stochastic Gradient Descent (SGD) was used as the optimizer; under the above settings, we initialized and pre-trained the ResNet 18 layer convolutional network to obtain a pre-trained model.

## 4. Experimental Results

To verify the effectiveness of the proposed framework, we trained on the dataset and used different metrics as metrics to highlight the performance. The experimental platform adopted the Ubuntu 18.04 system, and the algorithm model was built based on the open-source deep learning framework PyTorch. The hardware was Intel (R) Xeon (R) CPU E5-2620 v4@2.10 GHz CPU, 16 GB of RAM, NVIDIA GeForce GTX 1080 Ti GPU 4×4 . Python 3 is the programming language used.

### 4.1. Video Pre-Processing

**Figure 5:**
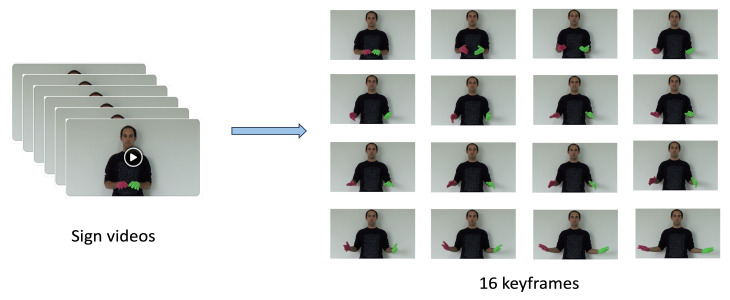
The diagram illustrates the process of data processing.

A video is a time series that is not used as raw input. In the early stage, video data need to be processed as shown Figure 5. In the video sequence, we clip the video into segments, each containing 16 key image frames. In order to increase its richness, we randomly crop and batch normalize keyframes, which can eliminate other redundant information and better express the extracted features. RGB is the most intuitive mode, and we adjust the keyframe size to 128×128 , with each video clip consisting of a 16 frame sequence. The size of the video clip is 16×128×128 .

### 4.2. Argentinie Sign Language Dataset

This experiment used the Argentine Sign Language dataset (LSA64) collected by the National University of La Plata [33]. LSA64 contains 64 categories of daily sign language words, each recorded by 10 participants, with each participant recording 5 times, each containing 50 sign languages. Examples of the categories in our dataset include the words ‘Bright’, ’Photo’, ’Music’, etc. Some commonly used sign language symbols, including verbs and nouns, were selected for the dataset. The entire sign language dataset contains 3200 videos. The database was recorded by inexperienced signers in two groups. In the first group, 23 one-handed gestures were recorded in an outdoor natural environment. In the second group, in an indoor environment, a synthetic light source was used to provide illumination differences between sign languages. A total of 41 gestures were recorded, including 23 double gestures and 19 single gestures; each performer is dressed in black with a white wall as the background, standing or sitting to perform the gestures. In order to better demonstrate the trajectory characteristics of sign language, each performer wears a pink glove on their right hand and a fluorescent glove on their right hand. Some sign language is performed with the right hand, and some with both hands simultaneously. During the recording process, the same distance and height were ensured. The pixel size of the recorded video data is 1920×1080 , containing 60 frames per second. With the purpose of verifying the generalization ability of the algorithm for non-specific individuals and comparing it with those of other methods, this study selected samples from 8 sign language learners as the training set, and the remaining 2 foreign language learners as the test set. Figure 1 shows a sample of LSA64 [34].

### 4.3. Metrics

In experimental classification results, accuracy is one of the most common classification evaluation indicators used to measure the accuracy of the classifier, which refers to the proportion of correctly classified samples to the total sample. In this adaptation, we used accuracy as the measurement indicator, but during the training process, we also obtained the F1 score, precision, and recall, which require four results to calculate, including the true positives ( TPs ), true negatives ( TNs ), false positives ( FPs ), and false negatives ( FNs ). The accuracy can be obtained using the following formula:

(11) $$\begin{array}{c} Acc=\frac{TP+TN}{TP+FP+TN+FN} \end{array}$$

The F1 score can be obtained using the following formula:

(12) $$\begin{array}{c} F_{1}=2*\frac{precision*recall}{precision+recall} \end{array}$$

Precision refers to the proportion of positive samples in the positive examples determined by the classifier. The precision can be obtained using the following formula:

(13) $$\begin{array}{c} Precision=\frac{TP}{TP+FP} \end{array}$$

Recall refers to the proportion of predicted positive cases to the total positive cases. Due to the need to use the recall function when calculating the F1 score, its calculation formula is as follows:

(14) $$\begin{array}{c} Recall=\frac{TP}{TP+FN} \end{array}$$

### 4.4. Implementation Details and Parameters

The pre-trained model mentioned above was used to freeze the weights of the pre-trained model. The pre-trained model was trained on ImageNet with 1000 categories, and the fully connected layer contained 1000 prediction classes. When training on our dataset, we retained the parameters of the pre-trained hidden layer and modified the parameters of the fully connected layer, and its output was the input of the LSTM network. For specific setting parameters, we utilized the video clip as the input for a better comparison with other methods and considered the temporal continuity of sign language words. A video clip contains 16 frame sequence as the data input for each training session. Each frame was randomly cropped to 128×128 to reduce the training consumption and overfitting issues, and the training and validation sets had the same input size to better achieve validation classification results. ReLU was used as a hidden activation function. The batch size of the network was set to 8, 16, and 32. Adam was used to optimize the model in the optimizer selection, with an initial learning rate of 0.0001 and weight decay of 0.0005. The adaptive learning rate adjustment method [35] (Reduce LR On Plateau) was used to automatically change the learning rate; its optimal threshold was set to 0.0001, and the number of epochs, with no improvement in tolerance indicators, was set to 5. When designing the loss function, we used a label smoothing cross-loss function to mitigate the impact of incorrect labels. The iteration period for each experiment was 50. With these experimental settings, we tried using different batch sizes and selected video clip sizes, as well as learning rates and optimizers, through continuous experiments. Based on the hyperparameters used in this study, we can obtain better results. The parameters are simplified, as shown in Table 2.

### 4.5. Results and Analysis

In this study, our proposed framework was validated on the LSA64 dataset. There were a total of 3200 sign language videos in 64 categories in the database, each containing 50 sign languages. We divided the dataset into training and validation datasets. Among them, training data accounted for 80%, which is 40 sign languages for each category, 2560 videos in total; the validation data accounted for 20%, which is 10 sign languages for each category, 640 videos in total. We set different batch sizes of 8, 16, and 32, and used the F1 score, precision, and accuracy measurement functions to obtain the accuracy of the validation set.

Table 3 shows the results, and we can see from the table that the batch size setting has little effect on the training convergence effect. As the number of epochs increases, the training accuracy gradually stabilizes, reaching a stable value around 20 epochs. In addition, we observed that setting 30 epochs can also achieve good results.

**Figure 6:**
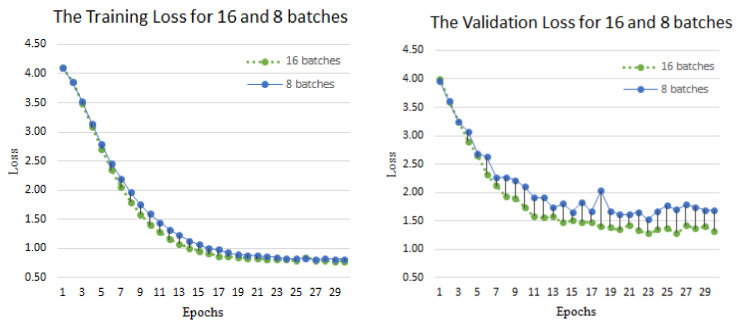
Training loss and validation loss on LSA64 dataset. The left shows that the loss of the training gradually decreases with the increase in iterations. The right shows the loss value of the validation gradually decreasing with the increase in epochs.

Figure 6 shows the training process on the Argentine Sign Language dataset. The loss indicates the convergence speed and stability of the model. We compared the training loss and validation loss of batches 8 and 16 at 30 epochs, respectively. From the figure, it can be seen that the fusion of the pre-train ResNet and LSTM network framework had a good convergence efficiency in the recognition process. It gradually stabilized at 23 epochs, with a training loss value of around 0.8 and a validation loss value of around 1.0. The reason for this situation is that the number of verified sign language datasets is too small. There was no vanishing gradient during the training process, and the use of the pre-trained model better demonstrated that our proposed method can reduce the training time while ensuring a good recognition accuracy, thereby ensuring good robustness of the model.

**Figure 7:**
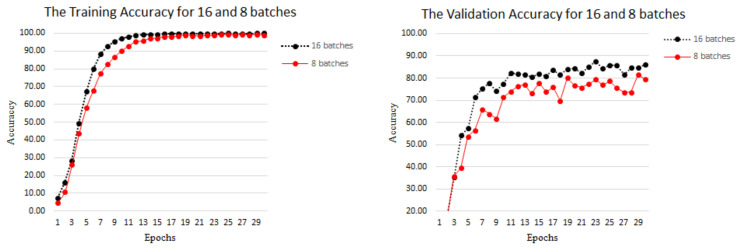
Training accuracy and loss on LSA64 dataset. The left shows that the accuracy of the training gradually increases with the increase in iterations. The right shows the loss value of the training gradually decreasing with the increase in iterations.

In Figure 7, we compare the training accuracy and validation accuracy, and the accuracy better reflects the model efficiency and usability. During the training process, the training accuracy gradually approached 100%, and the model stabilized at 19 epochs. In the subsequent training process, the training model showed a fitting phenomenon, and the model achieved a recognition rate of 100%. During the validation training process, the validation accuracy of 16 batches was higher than that of 8 batches, reaching a maximum of 86.25%. The reason for this situation is largely due to the input data. We divided each video into 16 frames of video segments, and during the training process, 16 batches were also grouped into one epoch. This network design can better extract features, but from Figure 7, the amplitude of this network’s variation is within a controllable range, which has little impact on recognition efficiency.

In comparing the method used in this article with other methods, as shown in Table 4, it can be seen that for the 3DCNN model, although it adds one-dimensional space for feature extraction in the time dimension, the effect is not very significant due to the fact that the 3DCNN model is based on continuous image convolution operations in processing time series. Sidig et al. [36] proposed using the ensemble k-nearest neighbor algorithm for recognition, but its effectiveness is not very obvious. In the CNN-LSTM model, the CNN is a simple neural network structure that cannot capture finer feature changes compared to residual network structures. Luqman et al. [37] used the logistic regression algorithm for sign language recognition, which is a relatively easy classification prediction algorithm. However, in sign language recognition with time series, the algorithm only achieved a recognition rate of 73.0%. Marais et al. [38] implemented the InceptionV3 and GRU network to achieve sign language recognition with a recognition rate of 74.22%.

After analysis, although the CRNN model, composed of a traditional CNN and LSTM, can extract spatiotemporal features from sign language videos, the accuracy of the sign language video recognition is affected by the vanishing or exploding gradients that traditional CNNs may experience as the network hierarchy deepens. So, in this article, using ResNet 18 instead of the traditional CNN can not only solve the problem of vanishing and exploding network gradients but also extract deeper feature information to improve the accuracy of network recognition. In addition, in order to better extract the spatial feature information of sign language and improve the model performance, this study used an LSTM to learn long-term features of the time series. The network can remove redundant information, thereby improving the accuracy and generalization ability of the model. In the end, we achieved an accuracy rate of 86.25% on the dataset, which better demonstrates the effectiveness of our proposed method.

## 5. Conclusions

On the basis of implementing video classification using two-dimensional networks, a series of factors that affect the stability of neural networks, such as gradient explosion and vanishing, are addressed; in time series, not only should the short-term feature space be considered, but the long-term feature space cannot be ignored. Long-term time series can highlight global features and capture the correlation between sequences. This article proposes a dynamic video sign language recognition method based on the fusion network of ResNet and LSTM, which achieved an accuracy of 86.25%, F1 score of 84.98%, and precision of 87.77%. The ResNet network was used as the skeleton model to learn the deep level feature information of dynamic sign language. The LSTM can obtain the feature information of long-term sequences and eliminate some redundant information. In the framework of this study, we also utilize the advantages of pre-trained models to improve the training efficiency and further enhance generalization in sign language recognition tasks. Finally, the performance of the model was validated on the LSA64 dataset, and the results show that the accuracy and reliability of the model were high, verifying the usability and effectiveness of the modified model. Although the performance of the model in this article is good, future work can focus on how to better learn spatial features, such as adding attention mechanisms and capturing the correlation of video sequences. In addition, multi-pose fusion feature extraction is also worth studying.

## Figures and Tables

**Table 1:** The parameters of the ResNet network.

Layer Name	Output Size	ResNet-18
conv1	112×112	7×7 , 64, stride 2
conv2_x	56×56	$\left[\begin{array}{c} 3\times 3,64\\ 3\times 3,64 \end{array}\right]\times 2$
conv3_x	28×28	$\left[\begin{array}{c} 3\times 3,128\\ 3\times 3,128 \end{array}\right]\times 2$
conv4_x	14×14	$\left[\begin{array}{c} 3\times 3,256\\ 3\times 3,256 \end{array}\right]\times 2$
conv5_x	7×7	$\left[\begin{array}{c} 3\times 3,516\\ 3\times 3,516 \end{array}\right]\times 2$
	1×1×1	GAP, FC layer with Softmax

**Table 2:** Training parameters.

Parameters	Setting
Framework	Pytorch
Epochs	50
Batch size	16
Frame size	128×128
Number of frames extracted per video	16
Number of features extracted per frame	RGB
Optimizer	SGD

**Table 3:** The validation accuracy for different epochs and batch sizes on the LSA64 dataset.

Epochs	Batch Size	F1 Score	Precision	Accuracy
10	8	69.81%	76.30%	72.66%
10	16	75.66%	82.79%	77.50%
10	32	72.64%	77.13%	75.31%
20	8	81.28%	84.59%	82.97%
20	16	83.01%	86.61%	84.38%
20	32	78.11%	80.68%	80.47%
30	8	81.19%	83.88%	83.12%
30	16	84.98%	87.77%	86.25%
30	32	79.29%	82.07%	81.88%
40	8	81.98%	85.37%	84.38%
40	16	83.20%	86.10%	84.69%
40	32	81.13%	85.89%	82.81%
50	8	80.60%	83.94%	82.19%
50	16	83.89%	85.95%	85.94%
50	32	82.19%	83.22%	84.22%

**Table 4:** Comparison of accuracies of different methods on LSA64 dataset.

Method	Modality	Classifier	Acc
3DCNN [39]	RGB	3DCNN	63.4%
Ensemble KNN [36]	RGB	KNN	64.0%
CNN-LSTM [37]	RGB	CNN and LSTM	72.4%
Logistic Regression [40]	RGB	Logistic Regression	73.0%
InceptionV3–GRU [38]	RGB	InceptionV3 and GRU	74.22%
ResNet LSTM (ours)	RGB	ResNet and LSTM	86.25%

## Data Availability

The experimental data are available at https://facundoq.github.io/datasets/lsa64/ (accessed on 1 August 2023).
